# Lignin Modification Supported by DFT-Based Theoretical Study as a Way to Produce Competitive Natural Antioxidants

**DOI:** 10.3390/molecules24091794

**Published:** 2019-05-09

**Authors:** Liga Lauberte, Gabin Fabre, Jevgenija Ponomarenko, Tatiana Dizhbite, Dmitry V. Evtuguin, Galina Telysheva, Patrick Trouillas

**Affiliations:** 1 Latvian State Institute of Wood Chemistry, Dzerbenes Str. 27, LV-1006 Riga, Latvia; roze@edi.lv (L.L.); jevgenijaponomarenko@inbox.lv (J.P.); lcl@edi.lv (T.D.); 2 INSERM UMR 1248, Université de Limoges, Faculté de Pharmacie, 2 rue du Docteur Marcland, 87025 Limoges Cedex, France; gabin.fabre@gmail.com; 3 CICECO/University of Aveiro, Campus Universitário de Santiago, 3810-193 Aveiro, Portugal; dmitrye@ua.pt; 4 Regional Centre of Advanced Technologies and Materials, Department of Physical Chemistry, Faculty of Science, Palacký University, tř. 17 listopadu 12, 771 46 Olomouc, Czech Republic

**Keywords:** lignins, antioxidant activity, molecular rationalization, modification, stabilizers for polymers

## Abstract

The valorization of lignins as renewable aromatic feedstock is of utmost importance in terms of the use of sustainable resources. This study provides a deductive approach towards market-oriented lignin-derived antioxidants by ascertaining the direct effect of different structural features of lignin on the reactivity of its phenolic OH groups in the radical scavenging reactions. The antioxidant activity of a series of compounds, modeling lignin structural units, was experimentally characterized and rationalized, using thermodynamic descriptors. The calculated O–H bond dissociation enthalpies (BDE) of characteristic lignin subunits were used to predict the modification pathways of technical lignins. The last ones were isolated by soda delignification from different biomass sources and their oligomeric fractions were studied as a raw material for modification and production of optimized antioxidants. These were characterized in terms of chemical structure, molecular weight distribution, content of the functional groups, and the antioxidant activity. The developed approach for the targeted modification of lignins allowed the products competitive with two commercial synthetic phenolic antioxidants in both free radical scavenging and stabilization of thermooxidative destruction of polyurethane films.

## 1. Introduction

The lignocellulosic plant biomass has appeared a promising alternative to the fossil resources because of its abundance, renewability, versatility, and environmental safety. Lignin is one of the main components of plant biomass. However, unlike the polysaccharides, only a little amount of lignin is exploited to produce biobased chemicals so far [1,2,3,4,5,6]. 

Lignins are arranged as amorphous network polymers, as a result from the dehydrogenative radical polymerization of monolignols (p-coumaryl, coniferyl, and sinapyl alcohols), containing divers functional groups and reactive sites, e.g., phenolic, aliphatic, or carboxylic OH groups, as well as substituted aromatic sites or C=C bonds [7,8,9]. Due to stereo-hindered phenolic OH groups, lignins can be valorized as prospective antioxidants. Low toxicity and biodegradability properties of natural lignin-derived antioxidants make them a promising alternative to the synthetic analogs [10,11,12,13] in food, cosmetic, and pharmaceutical applications [10,11,14,15]. Applications of lignin-based antioxidants in composite materials have also been recognized [12,16,17,18]. Lignin functionalities exhibits effective radical scavenger effect to prevent autoxidation and depolymerization of cellulose in pulps and papers [19,20]. The incorporation of lignins into synthetic polymer systems can prevent them from excessive photo- and thermal oxidation. For example, the introduction of 0.5–10% (*w*/*w*) of lignins, isolated from the byproducts of the prehydrolysis of birch wood in polypropylene or recycled polypropylene, significantly increases its stability, in a synergic way with the commercial Irganox 1010 antioxidant [21]. 

The main factors which limit the commercial use of lignins as antioxidants are their high structural variability and variation in chemical composition, as well as the absence of clear guidance for structure–activity relationships, which would allow the control of their antioxidant activity. Some recent technological developments propose to separate lignin molecules into fractions with low polydispersity and well-defined properties [22,23,24,25]. Among such processes, solvent fractionation of technical lignins is an effective method allowing to select lignin antioxidants by regulating molecular mass, polarity, compatibility to a given substrate, and number of phenolic OH and other functionalities [22,23,26,27,28,29,30,31,32]. Despite the success of fractionation to enhance the antioxidant response, the lignin-derived antioxidants still cannot compete with commercial phenolic antioxidants [33,34,35].

Due to the incredibly complicated and irregular structure of lignins, the measure of their antioxidant capacity does not allow to evaluate the contribution of each structural fragment on this activity [36,37,38]. The constitution of a library of lignin fragments has enabled experimental studies of the structure–antioxidant activity relationship, which, for the first time, included some features of the side-chain structure of the phenylpropane subunits revealing major structural descriptors [33,34]. To improve the predictive character of these structure–activity relationships, the experimental data can also take advantage of theoretical chemistry data on various phenolic compounds [39,40,41]. A joint experimental and theoretical approach sounds particularly adapted to solve the problems of lignin chemistry and to develop the methods of lignins modification for enhancing of their antioxidant activity. Based on the previous experimental studies made for a diversity of technical lignins, a series of low-molecular weight phenolic fragments have been chosen, considered as being sufficiently representative to rebuild the response of the “lignin puzzle.”

The aim of the present work was to obtain new quantitative information about the direct contribution of the different substituents on the side-chains of lignin structural units and the linkages between these units on the capacity of phenolic OH groups to scavenge free radicals and to develop the approach for lignins modification to enhance their antioxidant potential. Lignin modification was made based on data obtained by joining density functional theory (DFT) calculations and experimental measurements. Such targeted modification supports promotion of market-oriented natural antioxidants from renewable sources. The sulfur-free lignins isolated by the soda delignification from different plant sources and their fractions were examined as prospective raw materials. The antioxidant activity of lignin products was compared to that of commercially available phenolic antioxidants in terms of stable free radical scavenging activity and the ability to reduce the thermooxidative destruction of polyurethane films. Unlike commercial phenolic antioxidants, which significantly inhibit the biodegradation of the composite materials, antioxidants derived from soda lignins are not only biodegradable, but they can also catalyze the biological decomposition of the material into which they are introduced [13].

## 2. Results and Discussion

**Figure 1:**
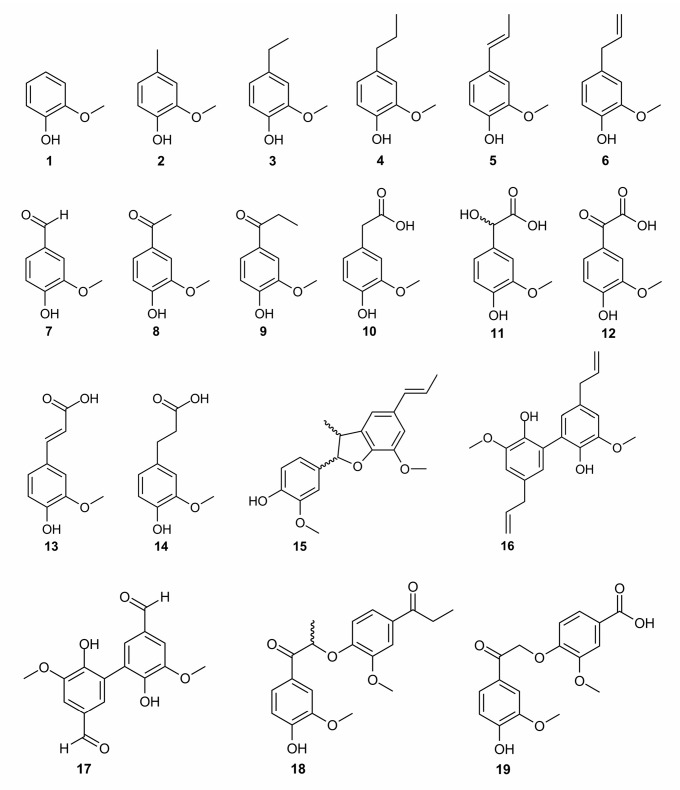
Chemical structures of the lignin model compounds.

The antioxidant activity of lignins is mainly attributed to phenolic OH groups whose reactivity in redox reactions is modulated by OCH_3_ groups [20,42,43]. This study has been focused on the contribution to antioxidant activity of the guaiacyl phenylpropane unit with different substitution patterns including side-chains and bonds between the structural units, which are insufficiently characterized so far. Accordingly, the antioxidant activity of 19 lignin-derived structural units was evaluated (Figure 1), all bearing a guaiacyl moiety with various structural modulations, being considered as representative of lignin structural fragments [34]. A contribution of each of these structures to the antioxidant activity was examined to develop the most appropriate method for modifying lignin to enhance its antioxidant activity. 

**Figure 2:**
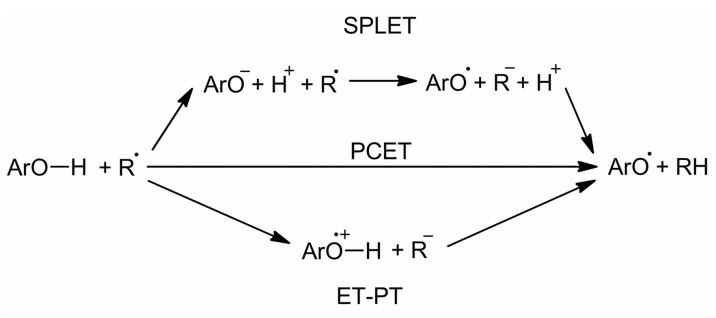
Different mechanisms for hydrogen atom transfer from polyphenols (ArO–H).

To establish a reliable structure–activity relationship (SAR), the related mechanism of free radical scavenging should be considered. The basic antioxidant mechanism of polyphenolics is the direct ROS (reactive oxygen species) scavenging by hydrogen atom transfer (HAT) mainly from phenolic OH groups (Figure 2). HAT may proceed either via (i) proton-coupled electron transfer (PCET), which is the concerted electron and proton transfer from the polyphenol to the free radical, (ii) or by sequential proton loss–electron transfer (SPLET), which is electron transfer from the deprotonated form (mainly phenolate or carboxylate in the presence of carboxylic acid moieties). Electron transfer directly from the polyphenol (electron transfer then proton transfer, ET-PT) is unlikely [41], being much more favored from the deprotonated forms (SPLET).

Disregarding the mechanism involved, the free radical scavenging by polyphenols is mainly driven by bond dissociation enthalpies (BDEs) of phenolic O–H. This descriptor is indeed directly related to the thermodynamic balance of the scavenging process, which is similar for both PCET and SPLET mechanisms. Therefore, BDE is a global descriptor of the antioxidant activity. In turn, ETE is a descriptor more specific for SPLET: it has no relevance under apolar and acidic medium conditions, but it may be seen as a minor descriptor under polar and neutral to alkaline conditions i.e., when SPLET may proceeds. The calculated BDE values varied from 77.6 kcal.mol^−1^ (typical for highly-reactive phenolic antioxidants) to 86.9 kcal.mol^−1^ (characteristic of low antioxidant activity), as shown in Table 1. The scavenging of the DPPH^•^ (2,2-diphenyl-1-picrylhydrazyl) and ABTS^•+^ (2,2’-azino-bis(3-ethylbenzothiazoline-6-sulphonic acid) free radicals often correlates with these thermodynamic descriptors [44,45,46] Conversely to BDE, the higher the radical deactivation index (RDI) value, the higher the antioxidant activity. Wide variations of RDI values were observed, ranging from 0.02 to 1.72 and from 0.16 to 2.39 in DPPH^•^ and ABTS^•+^ assays, respectively (Table 1). Assuming the RDI = 1.00 for guaiacol (**1**) as a reference (Table 1), RDI values lower or higher than 1.00 indicated a decrease or increase, respectively, in reactivity of the guaiacyl phenolic OH group. 

BDE values followed a trend similar to the RDI values of DPPH^•^ and ABTS^•+^, as evidenced by regression coefficients (R^2^) of 0.63 and 0.48, respectively. The regression analysis by excluding extreme cases (i.e., compounds **5** and **16**) nearly reached an acceptable linear correlation (R^2^ = 0.89 and 0.72, respectively). This confirms the relevance of BDE as the major descriptor of the antioxidant activity, as already seen for DPPH^•^ scavenging activity of flavonoids [44,45,46].

### 2.1. Role of the Alkyl Substituents

**Figure 3:**
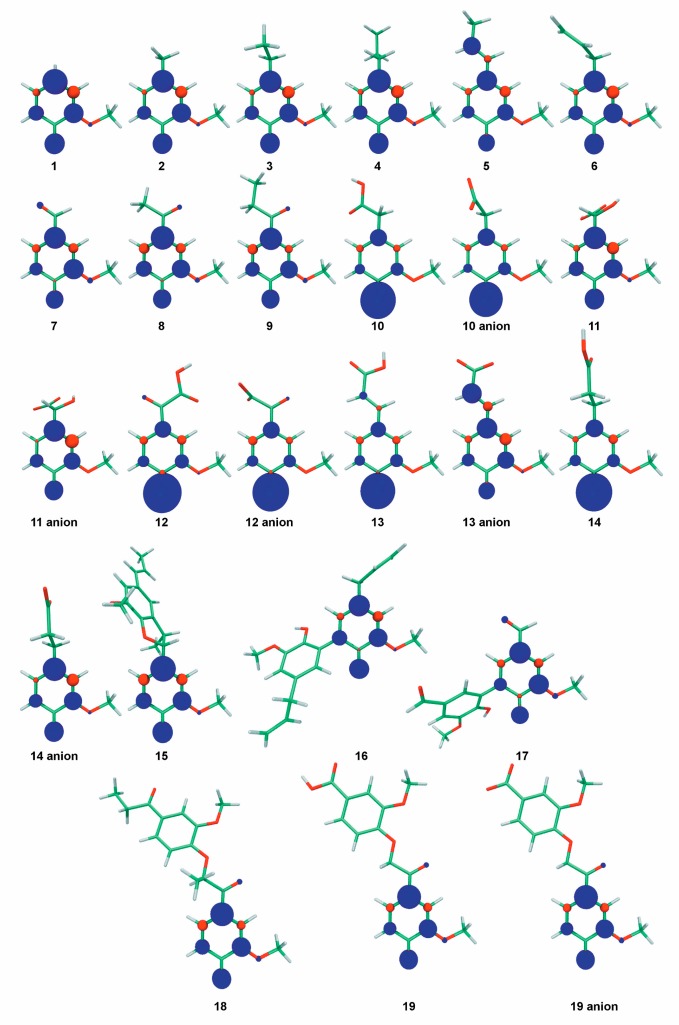
The spin density distribution of the aryloxyl radicals formed from the OH groups of the lignin-modeling structures.

With a BDE of 82.4 kcal mol^−1^, the unsubstituted guaiacol (**1**) is an efficient DPPH^•^ scavenger (Table 1). Compounds **2**, **3**, **4**, and **6** (creosol, ethylguaiacol, propylguaiacol, and eugenol, respectively) exhibited higher antioxidant activity in both DPPH^•^ and ABTS^•+^ assays, as seen from the RDI value higher than 1.00 (Table 1). This result agrees with the corresponding BDE values, which are lower than 82.4 kcal mol^−1^ for these three compounds (Table 1). This effect can be attributed to a positive inductive effect of the alkyl chain thus increasing the electrophilicity of this lignin structural unit. The length of saturated alkyl chains (methyl, ethyl or propyl) had negligible effect on BDEs and DPPH^•^ scavenging activity. Compounds **1**–**4** exhibited RDI values higher than 1, meaning that more than one radical can be scavenged per OH group. This also means that these guaiacol derivatives may form oxidative products (e.g., dimers) also behaving as efficient scavengers. The spin density distribution of the aryloxy radicals formed after HAT from the guaiacyl moieties highlighted multiple reactive sites for the further dimerization reactions in solution (Figure 3).

### 2.2. Role of π-Conjugation in the Side-Chain

Compounds **5** and **6** are special cases because of possessing delocalized π electrons in the side-chain at the *para* position to phenolic OH group. Both compounds differ from the location of the double bond (Figure 1). In **5**, the double bond is conjugated with the phenyl ring, allowing the radical delocalization after HAT over the phenyl ring and the side-chain. The longer the delocalization, the more stable the radical, and the lower the BDE. Compound **5** indeed exhibits a low BDE value of 77.7 kcal mol^−1^ (Table 1). This makes **5** efficient at scavenging both DPPH^•^ and ABTS^•+^ free radicals. Bortolomeazzi and coauthors evidenced that **5** may form oxidatively-induced biphenyl dimers, following fast kinetics [47]. These dimers appeared 90 times less active than **5** in terms of antioxidant efficacy, most probably because of the lowering of electrophilicity by acquiring the electron-accepting substituent in the 5th position of the guaiacyl unit (i.e., increasing BDE values). This is confirmed by the O–H BDE of 81.9 kcal mol^−1^ as obtained for the most abundant dimer dehydrodiisoeugenol (**15**), as seen in Table 1. Some contradiction between a very low BDE and the RDI value lower than **1** of isoeugenol is therefore rationalized by the quick formation of less active isoeugenol dimers. The high rate constant of dimerization may also cause quick self-termination between two isoeugenol radicals, explaining the fact that its stoichiometric factor is lower than **1** against DPPH^•^ [20]. The double bond in **6** is not conjugated with the phenyl ring, therefore decreasing the π-conjugated path compared to **5**. As a direct consequence, the BDE of the former is higher than that of the latter (81.0 and 77.7 kcal mol^−1^, respectively). However, surprisingly, **6** is more active than **2**–**4** at scavenging DPPH^•^ (Table 1). Dimers can also be formed by two eugenolyl radicals [47]. The most abundant dimer, dehydrodieugenol (**16**), is approximately twice as active as the monomer (**5**) in terms of free radical scavenging. The BDE of **16** is 80.1 kcal mol^−1^, confirming its better activity. As steric hindrance may prevent planarity of the phenyl rings and extension of the π-conjugated path in the dimer, the increase in activity is mainly due to the presence of two phenolic OH groups, each one being prone to HAT and to efficient scavenge more than one free radical per one phenolic group. Additionally, **16** reacts quicker with DPPH^•^ than **6** [47]. The **6** is therefore able to scavenge free radicals multiple times due to the formation of dimers, thus explaining the difference between its high BDE and the stoichiometric factors against DPPH^•^.

Surprisingly, both **5** and **6** showed a similar and efficient antioxidant activity against ABTS^•+^ (RDI values 1.45 and 1.44 for **5** and **6**, respectively). This can be rationalized by the kinetically preferential SPLET mechanism in water. Both compounds exhibit low ETE values (105.7 and 107.3 kcal mol^−1^, respectively), which correlate with their efficient antioxidation activity in water against ABTS^•+^.

### 2.3. Role of the Carbonyl Group in the α-Position of Guaiacyl Unit

Vanillin, acetovanillone, and propiovanillone (**7**–**9**) are guaiacol derivatives substituted with a chain bearing a carbonyl group in the α-position. These three compounds almost entirely lost DPPH^•^ scavenging activity (Table 1) and exhibited high BDE values ranging from 85.0 to 85.3 kcal mol^−1^. The carbonyl group, being highly electronegative, attracts the density of electrons, thus reducing the π-conjugation and the stability of the aroxyl radical (Figure 3).

The length of the side-chain (one to three carbon atoms for **7** to **9**) had no influence on BDEs and antioxidant activities. Interestingly, these three compounds still exhibited ABTS^•+^ scavenging activities (0.57, 0.54, and 0.45 for **7**, **8**, and **9**, respectively). As expected from the high BDE values, these activities were rather low, much lower than that of compounds **1**–**6**. The discrepancy between both assays may come from the thermodynamic balance of scavenging reactions. In the case of DPPH^•^, DPPH-H exhibits a BDE of 80.4 kcal mol^−1^ in polar solvent [42], hence HAT from compounds **7**–**9** to DPPH^•^ is thermodynamically unlikely, therefore forbidding almost all primary scavenging action. Moreover, compounds **7**, **8**, and **9** have pKa of 7.40, 7.81, and 7.98 (Table 1), reflecting acidity of the phenolic OH group. In buffered solution (pH = 7.35, see Experimental section), this corresponds to the presence of 44%, 11%, and 5% phenolates (deprotonated forms of the phenolic compounds), for the three compounds, respectively. Therefore, the SPLET mechanism may also proceed, as reflected by the ETE descriptors. Indeed, ABTS^•+^ electron affinity is −131 kcal mol^−1^ and the ETE values of **7**, **8**, and **9** are 121.4, 119.5, and 118.9 kcal mol^−1^, respectively.

### 2.4. Role of the Carboxylic Group in the β-Position of Guaiacyl Unit

Homovanillic acid (**10**) and vanillylmandelic acid (**11**) are guaiacyl derivatives substituted with a chain containing a carboxylic acid in β-position. The pK_a_ of both compounds are 4.41 and 3.43, respectively, in water solution corresponding to the carboxylic acid group [48]. Thus, in all assays assessed in water, such as the ABTS^•+^ assay, the monoanionic form must be considered for interpretation. In pure methanol, the carboxylic acid pK_a_ of both compounds is higher than **9** [49]. Therefore, concerning the DPPH^•^ assay performed in methanol, the protonated form is predominant. The antioxidant activity of **10** and **11** is similar to that of **1** (RDI of 1.18 and 0.98 for both compounds, respectively, as depicted in Table 1). Compound **10** appears slightly more active than **1** and **11**, in agreement with slightly lower BDE values (BDE of 82.0, 82.4, and 82.5 kcal.mol^−1^ for the three compounds, respectively); however, both from theoretical and experimental points of view these differences are almost insignificant. In **10** and **11**, the antioxidant constructive effect of the alkyl chain appears counterbalanced by the antioxidant destructive effect of the carboxylic acid moiety. It is worth noting that the hydroxyl group in the α-position of **11** has a slight detrimental effect. Low phenolic BDEs for the monoanionic forms (78.7 and 79.0 kcal mol^−1^ for **10** and **11**, respectively) rationalized the rather high ABTS^•+^ scavenging (RDI of 1.67 and 0.92, respectively). The slightly lower BDE of **10** may explain its somewhat better activity with respect to **11**, however this difference is not very significant. A better explanation is given by ETE, which appears slightly lower for **10** than for **11**. This confirms, at least partially, the contribution of SPLET mechanism, i.e., facilitated electron transfer from the carboxylated form to ABTS^•+^. Vanilglycolic acid (**12**) bears both the properties of a carbonyl group in α-position and a carboxylic acid in the β-position. This compound has no scavenging activity against DPPH^•^, which is fully supported by the high O–H BDE value (86.9 kcal mol^−1^). In water, **12** has two low pK_a_ (pK_a_1 = 1.60 and pK_a_2 = 7.54 [43]), meaning that in the ABTS^•+^ assay, this compound is mainly mono-deprotonated (carboxylate) and slightly bi-deprotonated (carboxylate and phenolate). The first ETE value (energy required to remove an electron from the carboxylate) is high thus making SPLET inefficient. The second ETE (energy required to remove an electron from the phenolate) is significantly lower, allowing SPLET towards ABTS^•+^. Therefore, the remaining ABTS^•+^ scavenging activity is most probably due to the small percentage of bi-deprotonated form of **12**.

### 2.5. Role of the Carboxylic Group in the γ-Position of Guaiacyl Unit

Ferulic acid (**13**) is a very well-known antioxidant polyphenol. Experimentally, it showed very good DPPH^•^ scavenging activity (RDI = 1.23), in agreement with previously reported values [50,51]. Its dihydrogenated analog dihydroferulic acid (**14**) exhibits the same DPPH^•^ scavenging activity. As for compounds with carboxylic acid moieties in β-position, these compounds exhibit low pK_a_ values in water (4.56 for **13**) but their pK_a_ values in methanol are expected to be much higher [49]. Therefore, **13** and **14** are most likely present in their protonated form in methanol. Hence, the similar BDEs of the protonated forms (81.8 and 81.1 kcal mol^−1^, respectively) rationalize similar DPPH^•^ scavenging activities for both compounds behaving by PCET. In water, both **13** and **14** are deprotonated from the carboxylic acid moieties (first pK_a_ value is 4.56 for **13** [48]). Therefore, the higher antioxidant activity of **13** against ABTS^•+^can be explained by the BDE of its deprotonated form (ferulate, BDE of 77.6 kcal mol^−1^) compared to **14** (dihydroferulate, BDE of 79.5 kcal mol^−1^). Additionally, the larger π-conjugated path (delocalization of electron density) in ferulate with respect to dihydroferulate also accounts for the higher activity of ferulate. In their respective radical forms, the spin density of the radical is only located on the phenyl ring for dihydroferulatoxyl radical, whereas it also spans the conjugated side-chain for ferulatoxyl radical (Figure 3). A higher delocalization of the spin density induces higher stability of the radical, thus in agreement with lower BDE and higher antioxidant activity. It should be noted that the effect of the π-conjugation is different in **13** and **14,** compared to **5** and **6,** due to the presence of the carboxylic moiety.

### 2.6. Effect of Dimerization

Compound **17** (divanillin) is the C5–C5 dimer formed from two vanillin molecules (Figure 1). It possesses two active symmetrical OH groups. Compounds **18** and **19** are arylether guaiacol dimers found in lignins. In both cases, the two guaiacyl units are β-*O*-4’linked. Thus, both compounds have only one active phenolic OH group. These three dimers exhibit carbonyl moieties in α-position on their *para* side-chains, so they share the same properties as their corresponding monomers (compounds **7** and **8**). Moreover, no additional π-conjugation arises from the dimerization process, even for divanillin in which steric hindrance prevents coplanarity of both phenyl rings. Therefore, these compounds show the absence or just weak activity against DPPH^•^ and ABTS^•+^, respectively, as confirmed by the high O–H BDE values (84.8, 86.0, and 86.5 kcal mol^−1^ for **17**, **18**, and **19**, respectively). It is worth noting that even though **19** has a carboxylic acid moiety that can be deprotonated in solution, it is located on the guaiacyl moiety without the active OH group so with almost no influence on the BDE (Table 1).

### 2.7. Quantification of the Direct Influence of the Each Structural Descriptor on the Antioxidant Activity of Lignins

Since BDE allows direct describing of the structure–antioxidant activity relationship, this parameter was chosen to quantify the contribution of each structural descriptor. For this purpose ΔBDE values were calculated among derivatives with or without of the corresponding chemical pattern (Table 2). The ΔBDE was calculated for each pair of compounds shown in Table 2 and the average ΔBDE value of the involved pairs of compounds was taken for each structural descriptor (Table 2). 

The presence of α-carbonyl group affects the reactivity of phenolic OH group much stronger in comparison to other structural descriptors, as can be seen by the biggest increase of BDE (highest ΔBDE value) (Table 2). This rationalizes that compounds containing α-carbonyl groups are almost inactive at scavenging DPPH^•^ radicals (e.g., RDI of compound **7** was 0.02, see Table 1). Because of this on/off effect, the ΔBDE of α-carbonyl groups was taken as a reference to normalize the impact of the other descriptors (Table 2). 

### 2.8. Experimental Evaluation of Lignin Antioxidant Activity with Respect to Impact of Different Structural Descriptors 

The quantitative impact of different structural descriptors (chemical substituents) on the reactivity of the guaiacyl OH group (Table 2) was used to rationalize the antioxidant activity of the oligomeric and polymeric lignin products and to find the most appropriate way for modification of technical lignins to obtain highly efficient phenolic antioxidants. Soda lignins used as a raw material in this study are heterogeneous both from point view of their chemical composition and the structure with a rather broad molecular weight distribution. Regarding the practical application of these antioxidants, numerous studies have highlighted the advantage of isolation of relatively uniform lignin fractions for these purposes, e.g., obtained by solvent fractionation. The most prospective approach is reported to obtain oligomeric fractions isolated from technical lignins by dichloromethane (CH_2_Cl_2_) [22,23,26,27,28,29,31,32,35]. Here, different soda lignins and their CH_2_Cl_2_ fractions were characterized in terms of the chemical composition, molecular weight distribution, content of functional groups, and other structural properties (Table 3).

**Figure 4:**
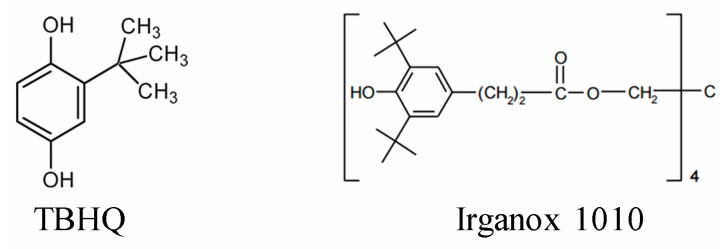
Chemical structures of the commercial antioxidants TBHQ and Irganox 1010, used as references.

Their antioxidant activity was examined and compared to that of the synthetic antioxidants TBHQ and Irganox 1010 (Figure 4), which are highly effective and widely used commercial phenolic antioxidants.

Despite numerous advantages of CH_2_Cl_2_-fractioning, including a significantly higher content of phenolic OH moieties, lower heterogeneity in terms of composition, and the molecular weight distribution in comparison to the parent lignins, a noticeable part of phenolic OH groups in CH_2_Cl_2_ fractions is inactive in radical scavenging reactions (i.e., RDI < 1, see Table 3). The antioxidant activity assay of the oligomeric lignin CH_2_Cl_2_ fractions shows that they are not competitive in comparison to the commercial phenolic antioxidants TBHQ and Irganox (Table 3). This behavior could be explained by the negative effect of the structural descriptors mentioned in Table 2 within the lignin structure, being the α-C=O the most suspicious. Hence, this proposition was verified by more detailed analysis of lignins and the corresponding CH_2_Cl_2_ fractions.

### 2.9. Rationalization and Enhancement of the Lignin Antioxidant Activity

**Figure 5:**
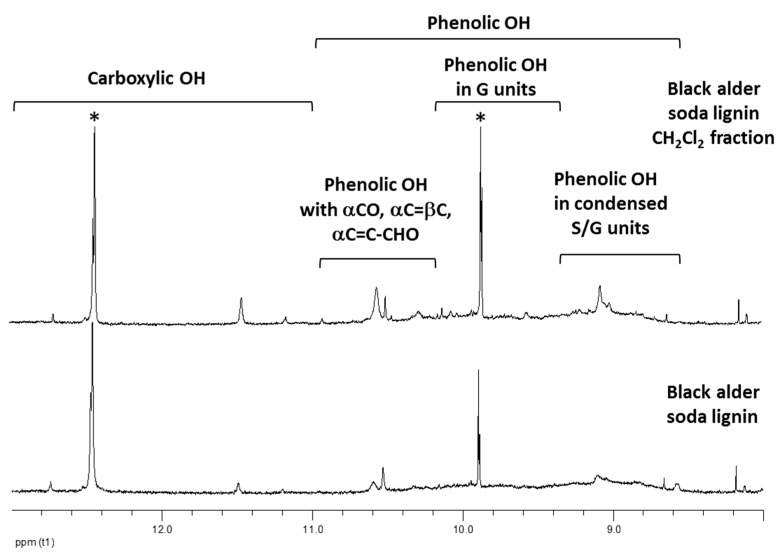
^1^H-NMR spectra (HMPT-d18, 298K) of parent black alder soda lignin and its fraction, extracted by CH_2_Cl_2_. Solvent contaminants peaks are marked by asterisk.

The analysis of lignins in strongly basic aprotic solvents allow to discriminate and quantify the phenolic hydroxyls in noncondensed and condensed structures [52,53]. As an example, the analysis of black alder lignin and its corresponding CH_2_Cl_2_ fraction is envisaged, but very similar features were find for other lignins in study. The ^1^H-NMR spectra in highly basic solution (HMPT-d_18_) show the difference between the black alder parent lignin and the corresponding CH_2_Cl_2_ fraction (Figure 5). 

The phenylpropane units containing phenolic OH revealed the characteristic resonances in the spectra range of 8.6 to 11.0 ppm [52]. CH_2_Cl_2_ fraction showed an enhanced content of α-carbonyl (αCO) (signal at 10.5–10.7 ppm [52]) and aldehyde groups (CHO) (signals at 10.2–10.4 ppm [52]) in the units containing the phenolic groups (Figure 5). They also have an increased content of carboxyl groups (signal at 11.46 ppm [52]) in comparison to parent lignin. According to the data, obtained by density functional theory (DFT) calculations (Table 2), the presence of these oxygen-containing groups in the side-chain, especially carbonyl groups, is the major reason for the inactivation of lignin CH_2_Cl_2_ fractions in radical scavenging reactions. The results of ^1^H-NMR analysis were confirmed by the Py-GC/MS, which shows the significantly higher yield of α-carbonyl group-containing compounds in the lignin derived products for CH_2_Cl_2_ fractions in comparison to the parent lignins (Table 3). 

**Figure 6:**
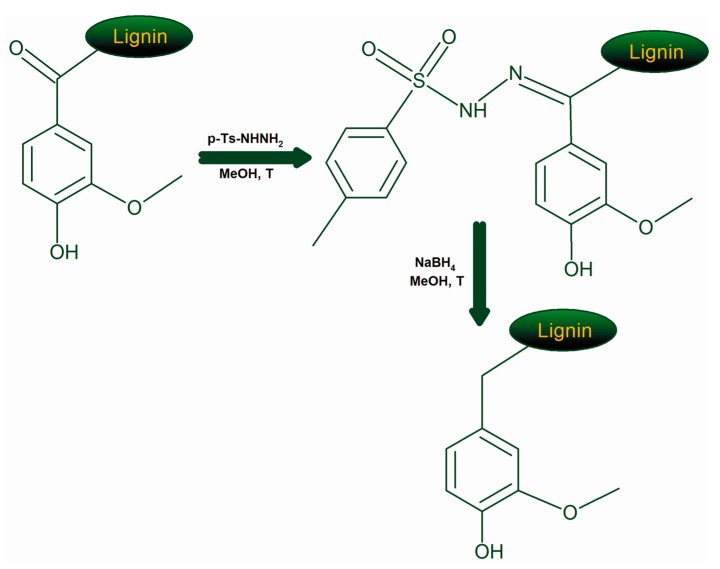
Reaction pathways used for the elimination of lignin carbonyl groups.

Due to the aforementioned structural features, the elimination of α-carbonyl is prone to increase drastically the antioxidant activity of lignin fractions. To tackle this hypothesis, the chemical reduction of α-carbonyl moieties was performed. The most commonly used reaction for the reduction of carbonyl groups of lignins is by using NaBH_4_; however, in such case the carbonyl groups are reduced to aliphatic OH groups which also have slightly negative effect on the antioxidant activity (Table 2). Instead, the chemical transformation of carbonyl groups into CH_2_ fragments would not only avoid the negative effect of the former but also bring the positive effect of the latter, see Table 2. Therefore, this modification of lignin products was performed according to the reaction sequence shown in Figure 6. 

**Figure 7:**
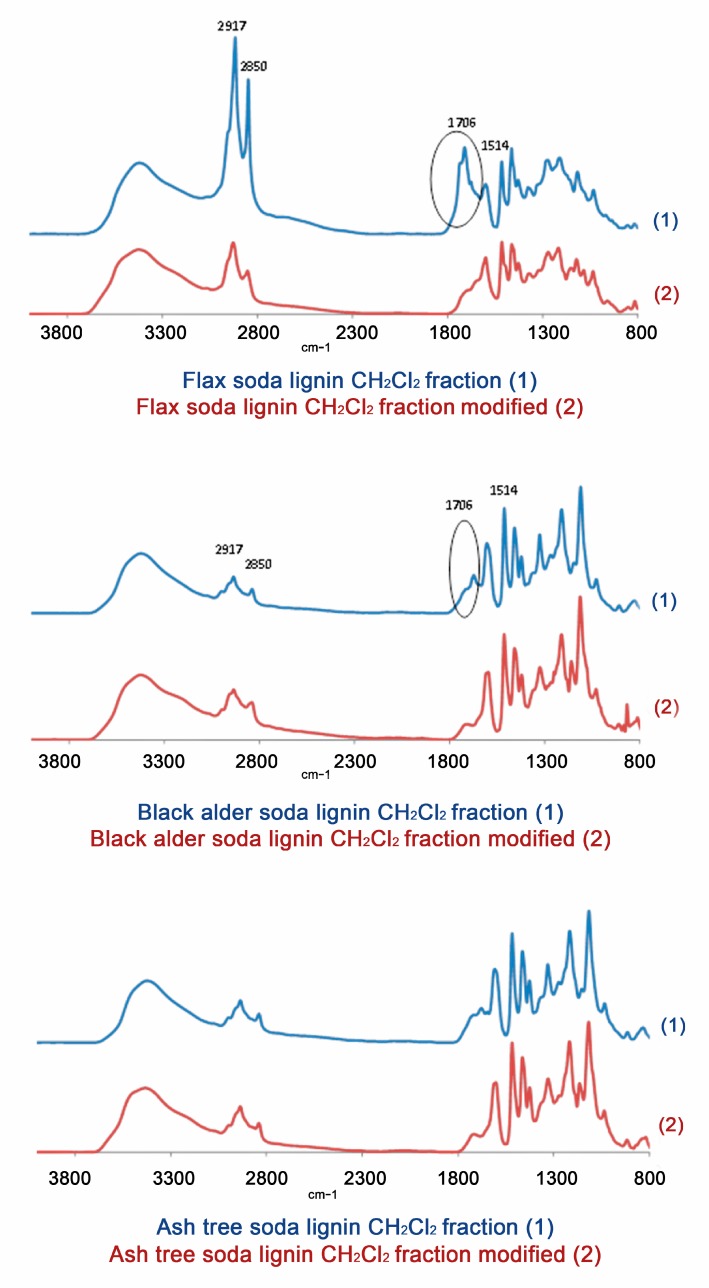
FTIR spectra of fractions obtained from soda lignins under study (**1**) before and (**2**) after their chemical modification (elimination of carbonyl groups).

The elimination of the carbonyl groups was also controlled by FTIR spectroscopy (Figure 7). The variation of the intensity of the band assigned to the carbonyl groups vibration in the normalized spectra of flax, black alder, and ash-tree soda lignin CH_2_Cl_2_ fractions after their modification decreased from 76.2 to 19.5%, from 29.7 to 11.3%, and from 28.0 to 13.0%, respectively (Figure 7).

**Figure 8:**
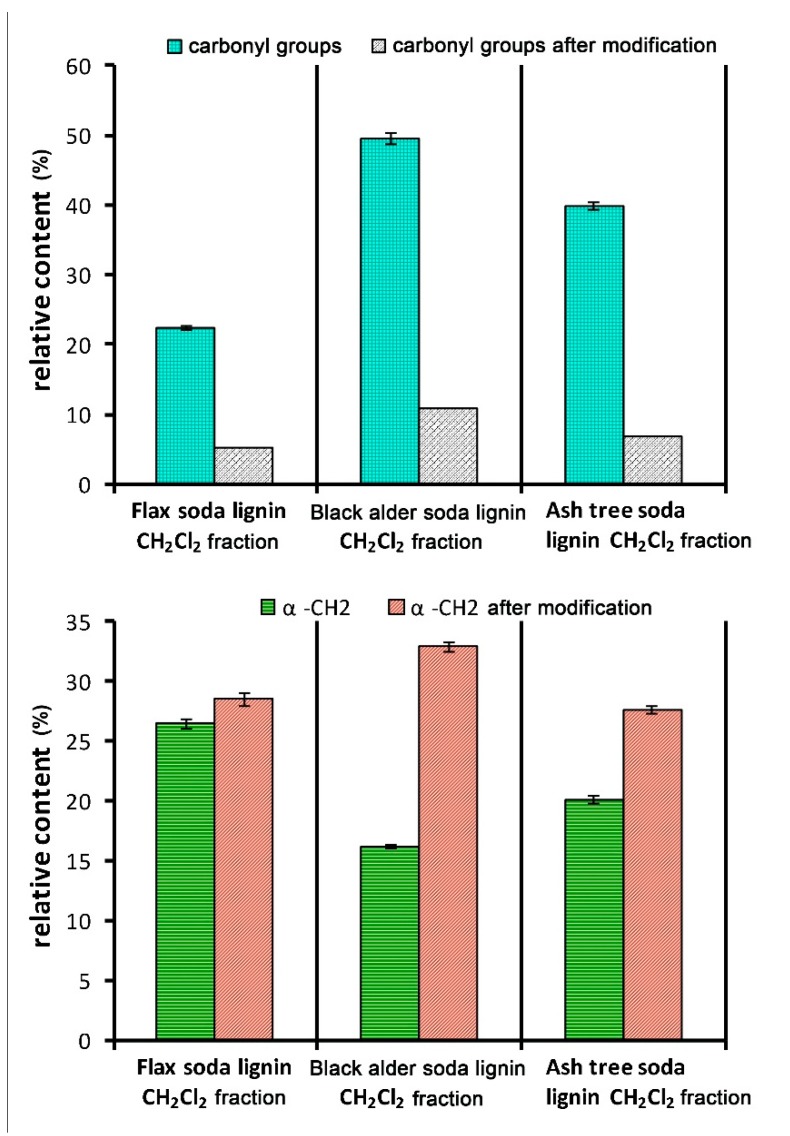
Change of the relative content of the compounds containing carbonyl groups (C=O) and –CH_2_ fragment at the α-position of side-chain (α-CH_2_) in the lignin-derived compounds (Py-GC/MS data), caused by the modification of the CH_2_Cl_2_ fractions of soda lignins.

The elimination of the carbonyl groups was also confirmed by Py-GC/MS showing a significant decrease in the yield of the lignin-derived compounds containing the carbonyl group with a simultaneous increase in the yield of the compounds containing α-CH_2_ fragments (Figure 8).

The decrease of the relative content of phenolic OH groups conjugated with carbonyl groups (with respect to the total OH phenolic) was also confirmed using differential UV/Vis spectroscopy (Table 4).

As it was predicted, the elimination of carbonyl groups drastically increases the reactivity of lignins phenolic OH groups making them competitive with respect to the commercial phenolic antioxidants TBHQ and Irganox 1010 (Table 4).

### 2.10. Modified Lignin Fractions as Alternative Antioxidants in Polyurethane Films

Commercially available synthetic antioxidants inhibit significantly the biodegradation of polymeric materials. Conversely, lignins can stimulate their decomposition under natural environment after the end of their service life. Therefore, replacing synthetic antioxidants with natural ones would promote the production of environmentally friendly materials. For example, it was shown that the addition of lignin (4.2% to 9.3% in weight) in the poly(ethylene adipate) urethane films significantly improved their degradability by fungi enzymes (e.g., by laccase isolated from the *Aspergillus sp.*) [13].

**Figure 9:**
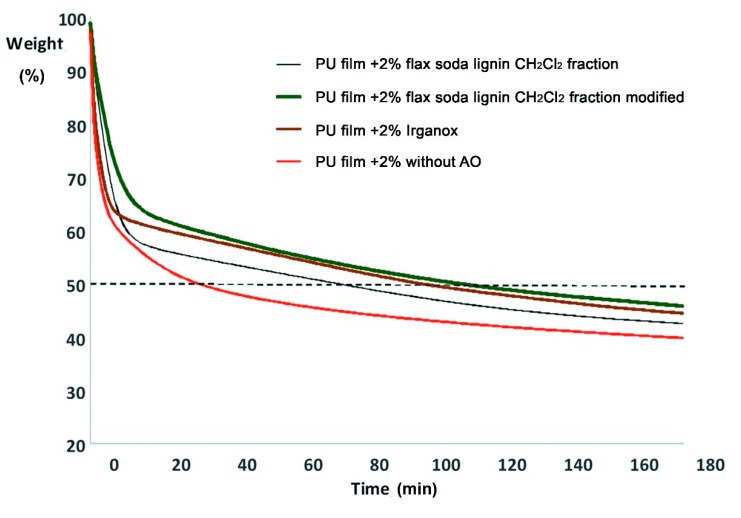
Kinetics of the thermooxidative degradation of polyurethane (PU) films (with and without addition of antioxidant).

In this work, the influence of different antioxidants on thermooxidative destruction of polyurethane (PU) films at 310 °C in air was studied for the demonstration purposes. Accordingly, flax soda lignin modification products were compared to Irganox 1010, a highly effective antioxidant against thermooxidative degradation of polyurethanes [54]. The kinetic curves of the thermooxidative degradation of the polyurethane films show that the addition of the antioxidant decreases the weight loss rate, especially in the first stage of thermooxidation destruction (Figure 9), which proceeds by radical mechanism [55].

Without antioxidant, 50% of the weight loss of the material is reached after 32.5 min of incubation. With 2% of flax soda lignin CH_2_Cl_2_ fraction 50% of the weight loss of the material is reached after 74.5 min of incubation. Adding 2% of Irganox or modified flax soda lignin CH_2_Cl_2_ fraction increases this time to 100 or 111.5 min, respectively (Figure 9).

The antioxidant activity was also assessed as integrals of the kinetic curves (Figure 9). The integral antioxidant activity as portion from the activity of Irganox was 0.6 for flax soda lignin CH_2_Cl_2_ fraction and 1.3 for modified flax soda lignin CH_2_Cl_2_ fraction. This indicated that the modified flax soda lignin CH_2_Cl_2_ fraction performed 30% better inhibition effect than Irganox. This confirms a highly efficient antioxidant activity of this lignin derivative, and outlines the promising application of lignins in environmentally friendly materials.

## 3. Materials and Methods 

### 3.1. Compounds, Modeling Lignin Structural Units

Model compounds **1**–**11**, **13**, **14**, and **17** (Figure 1) used in this study are commercially available:

**1.**: 2-methoxyphenol (guaiacol), ≥ 98%, Sigma Aldrich, CAS: 90-05-1

**2.**: 2-methoxy-4-methylphenol (4-methylguaiacol), ≥ 99%, Acros organics, CAS 93-51-6

**3.**: 2-methoxy-4-ethylphenol (p-ethylguaiacol), ≥ 98%, SAFC, CAS 2785-89-9

**4.**: 2-metoxy-4-propylphenol (4-propylguaiacol), ≥ 99%, Sigma Aldrich, CAS 2785-87-7

**5.**: 2-methoxy-4-propenylphenol (isoeugenol), 99%, Sigma Aldrich, CAS 97-54-1

**6.**: 4-allyl-2-methoxyphenol (eugenol), ≥ 98%, Sigma Aldrich CAS 97-53-0

**7.**: 4-hydroxy-3-methoxybenzaldehyde (vanillin), 99%, Sigma Aldrich, CAS 121-33-5

**8.**: 4’-hydroxy-3’-methoxyacetophenone (acetovanillone), 98%, Acros organics, CAS498-01-2

**9.**: 1-(4-hydroxy-3-methoxyphenyl)-1-propanone (propiovanillone), ≥ 95%, Bide Pharmatech, CAS 1835-14-9

**10.**: 4-hydroxy-3-methoxyphenylacetic acid (homovanilic acid), Acros organics, CAS 306-08-1

**11.**: (RS)-hydroxy(4-hydroxy-3-methoxy-phenyl)acetic acid (vanillylmandelic acid), 99%, Acros organics, CAS 55-10-7

**13.: ** (E)-3-(4-hydroxy-3-methoxy-phenyl)prop-2-enoic acid (trans-ferulic acid), 99%, Sigma Aldrich, CAS 537-98-4

**14.**: 3-(4-hydroxy-3-methoxyphenyl) propanoic acid (dihydroferulic acid), ≥ 96%, Sigma Aldrich, CAS 1135-23-5

**17.**: 6,6′-dihydroxy-5,5′-dimethoxy-[1,1′-biphenyl]-3,3′-dicarboxaldehyde (divanillin), Fluorochem, CAS 2092-49-1

Compound **12** (4-hydroxy-3-methoxyphenyl)(oxo) acetic acid (or vanilglycolic acid) was synthesized at the Latvian State Institute of Wood Chemistry by the oxidation of compound **8** by nitrobenzene in alkaline medium at 100 °C, using the method reported by Mottern [56] with minor modifications. Compound **8** (35 g), nitrobenzene (27 g), and NaOH (25 g) were mixed in 75 mL water at 100 °C for 24 h. Aniline and small amount of nitrobenzene were distilled with water vapor; the cooled solution was acidified, treated with active oxygen and filtered. The solution was cooled and compound **12** was crystallized. The amount of compound **12** was 34 g (85%) (T_melting_ = 133 °C) and its purity was 85%, as checked chromatographically (GC, gas chromatography). 

Compounds **18** and **19** (Figure 1) were synthesized by the methods described previously [57,58,59]. Their purity was 88 and 90%, respectively, as checked by GC.

### 3.2. High-Molecular Weight Lignins

The soda sulfur-free lignins were produced on industrial scale from annual (nonwood) plants using the patented lignin precipitation process applied to black liquor from soda pulping [60]. Soda lignin from flax (*Linum usitatissimum*) was kindly supplied by the Granit SA (Lausanne, Switzerland). 

The wood based soda lignins were obtained as a by-product from the black liquor of soda pulping (the IWC pilot equipment). The wood chips of black alder (*Alnus glutinosa*) and the ash-tree (Fraxinus excelsior) were pre-extracted with acetone to remove the extractives according to TAPPI standard T 280 pm-99. The residual content of the extractives was 0.15% on the oven-dried wood. The 280 g of chips were cooked in the digester with 2240 mL of 5% sodium hydroxide (Sigma Aldrich, Steinheim, Germany) which corresponds to an alkali charge of 42% based on the dry wood. Once the reactor reached the operating temperature of 160 °C, it was maintained for 3 h. The pulp was filtered, and the filtrate was acidified to pH 2 to precipitate the lignin part. Lignin was filtered off and washed with a hot water until neutral reaction. The obtained lignin was dried under vacuum at 40 °C. The yields of soda lignins from black alder and ash tree were ~20%.

Both industrial and laboratory lignins were used as raw materials for modification to obtain the highly effective lignin-derived antioxidants. The oligomeric fraction of lignin was obtained by fractionation with CH_2_Cl_2_ as described previously [61].

### 3.3. Computational Methods

The O–H bond dissociation enthalpy (BDE) of each phenol moiety (ArO-H) was calculated based on the enthalpies balance as follows.

BDE(ArO-**H**) = H^0^(ArO^•^) + H^0^(**H**^•^) – H^0(^ArO**H**) (1)

It is noteworthy that the lower the BDE, the easier the cleavage of the O–H bond, and the higher the antioxidant activity. Similarly, the electron transfer enthalpy (ETE) was calculated as follows.

ETE(ArO^−^) = H^0^(ArO^•^) – H^0^(ArO^−^) (2)

DFT calculations were performed with the B3P86 functional in Gaussian program as it was shown to accurately describe polyphenol BDEs [39,40,62,63]. The 6-31+G(d,p) basis set was used as providing very similar results compared to the larger and more computationally demanding 6-311+G(2d,3pd) basis set. Ground-state geometries were confirmed by a vibrational frequency analysis that indicated the absence of imaginary frequency. Enthalpies were calculated at 298 K and 1 atm. As many lignin fragments possess carboxylic acid moieties that can be deprotonated in aqueous media, all protonation states were evaluated. The solvent effect was considered using the integral-equation-formalism polarizable continuum model (IEF-PCM). Continuum models consider the molecular system embedded in a shape-adapted cavity surrounded by a dielectric continuum characterized by its permittivity (ε = 32.61 for methanol). Calculations in methanol reproduced the conditions of the DPPH^•^ assay. All calculations were performed with the Gaussian09^®^ software from Gauss Inc, USA [64].

### 3.4. Antioxidant Activity Assays (DPPH^•^, ABTS^•+^) 

Chemicals used for the antioxidant evaluation (DPPH^•^, ABTS^•+^ and solvents) were of analytical grade (Sigma–Aldrich). All solutions were prepared freshly before the measurements.

DPPH^•^ (2,2-diphenyl-1-picrylhydrazyl) was dissolved in methanol at a final concentration of ~10–4 mol L^−1^ and incubated in the dark at room temperature for 16 h. The exact concentration of DPPH^•^ in terms of absorbency at 515 nm was calculated from a calibration curve (A_515_ = 8.3 × C_DPPH_^•^ + 0.001).

A stock solution of ABTS (2 mM) was prepared in 50 mM phosphate buffered saline (PBS) made of 8.18 g NaCl, 0.27 g KH_2_PO_4_, 3.58 g NaHPO_4_ x11 H_2_O, and 0.15 g KCl in 1 L of distilled water. When necessary, the pH of the solutions was adjusted at 7.4 with 0.1 M NaOH. The ABTS^•+^ solution was produced by mixing 50 mL of ABTS stock solution with 0.2 mL of K_2_S_2_O_8_ (70 mM) aqueous solution. The mixture was kept in the dark at room temperature during hours 15 to 16 before use. To evaluate the antioxidant capacity, the ABTS^•+^ solution was diluted with PBS to reach the absorbance at 734 nm of 0.800 ± 0.030 [65]. The exact concentration of ABTS^•+^ was calculated from the calibration curve (A_734_ = 23.85 × C_ABTS_^•+^ − 0.007).

A 0.03 mL DMSO solution of the lignin or relative phenolic compounds was added to 3 mL of the prepared the DPPH^•^ or the ABTS^•+^ solution. Five different concentrations were used for each derivative. The decrease in DPPH^•^ or ABTS^•+^ concentration was followed at a 515 or 734 nm respectively until the reaction’s completion using Perkin Elmer UV/VIS spectrometer Lambda 650 equipped with a thermally controlled cell at 22 °C. All experiments were done in triplicate. The DPPH^•^ or ABTS^•+^ concentrations at steady state (i.e., primary reaction completed and no change in solution for most of compounds) were plotted as a function of the molar concentrations of the lignins or relative phenolic compounds. The antioxidant activity was expressed as a radical deactivation index (RDI) which corresponds to the number of deactivated free radicals per molecule of tested compounds in the case of lignin modeling compounds, or as the number of deactivated free radicals per phenolic OH group in the case of technical lignins and their fractions. The obtained results are shown as the average with a confidence interval (α = 0.05 level of significance). The RDI index is independent of the free radical concentration. The higher the RDI, the higher the free radical scavenging activity.

### 3.5. Characterization of the Polymeric and Oligomeric Lignin Products

#### 3.5.1. Molecular Weight Distribution 

The molecular weight distribution (MWD) of the parent lignins and the fractions obtained was analyzed by size exclusion chromatography (SEC) described in [66] using the Agilent 1100 system (PaloAlto, CA, USA). Lignin samples dissolved (1 mg mL^−1^) in the eluent—DMSO—were injected into the PolarGeL-L 300 × 7.5 mm column (Polymer Laboratories). The flow rate was 0.8 mL min^−1^, column was thermostated at 60 °C. A differential refractometer (RI) and a UV photometer (at λ = 254 nm) were used as detectors. For calibration, polystyrene standards were used in the range of 600 to 60,000 Da.

#### 3.5.2. Analytical Pyrolysis

The analytical pyrolysis (Py-GC/MS) analysis was performed using a Frontier Lab (Japan) Micro Double-shot Pyrolyser Py-2020iD (500 °C pyrolysis temperature, 600 °C s^−1^ heating rate) directly coupled with the Shimadzu GC/MS–QP 2010 apparatus (Tokyo, Japan) with capillary column RTX-1701 (Restek, Bellefonte, PA, USA), 60 m × 0.25 mm × 0.25 μm film (injector temperature 523 K, ion source 523 K with EI of 70 eV, MS scan range *m*/*z* 15 to 350, carrier gas helium at the flow rate of 1 mL min^−1^, and split ratio of 1:30. The mass of a sample was 1.00–2.00 mg. The oven program was 1 min isothermal at 333 K, then 6 K min^−1^ to 543, and finally held at 543 K for 10 min. The apparatus was modified by installation of the splitter of gas carrier flow Vitreous Silica Outlet Splitter VSOS (SGE, Australia) to operate FID and MS detectors simultaneously. The identification of the individual compounds was performed on the basis of GC/MS chromatogram using the Library MS NIST 147.LI13; whereas, the relative area of the peak of individual compounds was calculated using the Shimadzu software on the basis of GC/FID data. The summed molar areas of the relevant peaks were normalized to 100%. Relative peak areas calculated for pyrolysis products of different origin (lignins, carbohydrates, and lipophilic extractives) were used for assessment of the composition of the lignin samples.

#### 3.5.3. Spectroscopic Analyses

Fourier transform infrared (FTIR) spectra of lignins were recorded in KBr pellets by Spectrum One apparatus (Perkin Elmer) with 4 cm^−1^ resolution and 64 scans. ^1^H-NMR spectra of the non-acetylated lignin samples were recorded on a BrukerAvance 300 spectrometer (Bruker, Wissembourg, France) operating at 300.1 MHz and at 298K. The spectra were registered in the hexamethylphosphoroustriamide (HMPT-d18) using a sample concentration of 2.5% (*w*/*v*). Tetramethylsylane (TMS) was used as an internal standard (δ = 0.00). The relaxation delay was 3.0 s and about 300 scans were collected (90° pulse).

#### 3.5.4. Functional Analysis

The content of OCH_3_ groups in lignin samples was determined by the Viebock–Schwappach method [45]. The results were expressed on a dry weight and ash-free basis. The number of total and phenolic OH groups was determined, using ^1^H-NMR spectroscopy for the acetylated lignin samples [67]. ^1^H-NMR spectra were recorded on a Bruker Avance 300 spectrometer (Bruker, Wissembourg, France) operating at 300.1 MHz and at 298K. The spectra were registered in CDCl_3_ using typical sample concentrations of 2.5% (*w*/*v*). Tetramethylsylane (TMS) was used as an internal standard (δ = 0.00). The relaxation delay was 3.0 s and about 300 scans were collected (90° pulse). The number of phenolic OH groups with conjugated carbonyl groups was determined by differential UV/VIS spectroscopy. The lignin samples (10–15 mg) were dissolved in 10 mL dioxane. Two milliliters of the prepared solution was transferred in three 50-mL volumetric flasks. One of the flasks was filled with the buffer solution at pH 6, the second flask was filled with the buffer solution at pH 12, and the third flask was filled with 0.2 M NaOH. The optical density of the prepared alkaline solutions was measured against the optical density of the neutral solution (pH 6) at 300 nm and 360 nm, using Perkin Elmer UV/Vis spectrometer Lambda 650. (Waltham, MA, USA). Calculations of the number of phenolic OH groups were made as described elsewhere [59].

### 3.6. Modification of the Lignins by Reduction of Carbonyl Groups

The procedure describing the reduction of aldehyde and ketone functions to methylene groups, using the tosylhydrazone derivatives under very mild conditions [68] was adapted for lignin. The yield of the modified lignin was ~75%. Recrystallization from the aqueous methanol provides analytically pure product with the yield ~64%.

### 3.7. The Effect of Lignin Products on Thermooxidative Destruction of Polyurethane Films

Thermooxidative destruction of the model polyurethane (PU) elastomer (control and containing 2% of lignin sample films) was studied at 310 °C under isothermal regime by TGA in air atmosphere (flow rate 50 mL min^−1^) using the Metler Toledo Star System TGA/ADTA 851e device. A 8–10 mg sample was used. The PU elastomer films were obtained as described previously [33].

## 4. Conclusions

In this work, an integrated and multidisciplinary approach for the better understanding the antioxidant activity of lignins has been applied. By combining molecular modeling and antioxidant assays, the antioxidant properties of lignin phenylpropane units were rationalized. The calculation of O–H bond dissociation enthalpies (BDE) of characteristic lignin subunits allowed us to quantify the direct influence of key structural features on antioxidant activity of lignin. Importantly, the presence of carbonyl groups conjugated with phenolic units almost completely (98%) inactivated the antioxidant activity of phenylpropane units. Other structural features, such as aryl ether linkages or carboxyl groups in phenolic units, contributed to a reduction of the redox reactivity to ~30% or 16–33% (calculated as normalized ΔBDE), respectively, when compared to an unsubstituted analog. On the other hand, the presence of –αCH_2_– or –αCH_3_ fragments in the side-chains of phenylpropane units resulted in an increase of the redox reactivity of their phenolic groups by approximately 40% (calculated as normalized ΔBDE).

The antioxidant activity of soda lignins from flax, black alder and ash-tree was also examined. It was suggested that the decrease in heterogeneity and polydispersity, as well as a high content of phenolic OH groups in the oligomeric fractions of lignins make them good candidates in antioxidation applications. However, such undesirable structural features as high content of carbonyl or carboxyl groups at the alpha or beta positions of the propane chain, drastically decrease the antioxidant capacity of lignins over commercial phenolic type antioxidants. However, the proposed targeted modification of lignins fractions allowed a significant decrease in carbonyl groups conjugated with phenolic structural units, with a simultaneous increase in CH_2_ groups at α-position of the side-chains. This resulted in a drastic increase of the phenolic reactivity making their antioxidant activity competitive with the synthetic commercial antioxidants produced from fossil resources. These modified lignins were more effective in preventing the thermo-oxidation of PU films compare to the reference synthetic antioxidants, therefore appearing as very promising innovative environmentally friendly and biodegradable antioxidation agents.

## Figures and Tables

**Table 1:** Calculated O–H bond dissociation enthalpy (BDE), electron transfer enthalpy (ETE), and radical deactivation indexes (RDI) in DPPH^•^ and ABTS^•+^ assays of the lignin modeling compounds.

Number of the Compound	Lignin Modeling Compound	BDE (kcal mol^−1^)	DPPH^•^ RDI	ETE (kcal mol^−1^)	ABTS^•+^ RDI	pK_a_ in Water ^b^
**1**	guaiacol	82.4	1.00 ± 0.03	109.0	-	9.93
**2**	methylguaiacol	80.3	1.38 ± 0.03	105.7	0.92 ± 0.03	10.27
**3**	ethylguaiacol	80.5	1.35 ± 0.04	106.0	-	-
**4**	propylguaiacol	80.4	1.53 ± 0.02	106.0	1.58 ± 0.07	9.85
**5**	isoeugenol	77.7	0.90 ± 0.09	105.7	0.67 ± 0.05	9.89
**6**	eugenol	81.0	1.72 ± 0.07	107.3	0.96 ± 0.04	10.15
**7**	vanillin	85.3	0.02 ± 0.01	121.4	0.57 ± 0.03	7.40
**8**	acetovanillone	85.2	0.03 ± 0.01	119.5	0.54 ± 0.03	7.81
**9**	propiovanillone	85.0	0.05 ± 0.01	118.9	0.45 ± 0.04	7.98
**10**	homovanillic acid	82.0/78.7	1.18 ± 0.03	109.1/102.0	1.67 ± 0.02	4.41/ 10.52
**11**	vanillylmandelic acid	82.5/79.0	0.98 ± 0.02	111.3/103.3	0.92 ± 0.02	3.43/ 9.93
**12**	vanilglycolic acid	86.9/83.7	0.08 ± 0.01	125.7/116.7	0.26 ± 0.01	1.60/ 7.54
**13**	ferulic acid	81.8/77.6	1.23 ± 0.03	116.8/106.0	2.39 ± 0.07	4.56/ 9.39
**14**	dihydroferulic acid	81.2/79.5	1.23 ± 0.06	107.4/103.5	1.39 ± 0.04	-
**15**	dehydrodiisoeugenol	81.9	0.67 ± 0.02 ^a^	109.5	-	-
**16**	dehydrodieugenol	80.1	2.72 ± 0.03 ^a^	106.3	-	-
**17**	divanillin	84.8	n.d. ^c^	121.6	0.16 ± 0.03	6.16/ 10.07
**18**	dipropiovanillone	86.0	0.014 ± 0.01	122.0	-	-
**19**	acetovanillonylvanillic acid	86.5/85.6	0.02 ± 0.01	121.5/120.0	0.26	-

^a^ imported from Bortolomeazzi, et al. [47]; ^b^ imported from Ragnar, et al. [48]; ^c^ the compounds insoluble in the reaction medium.

**Table 2:** Quantitative evaluation of the impact of different structural descriptors (chemical substituents) on the reactivity of the guaiacyl OH group.

Structural Descriptor	Compounds with and without the Indicated Chemical Feature *	Average ΔBDE of Phenolic OH Group between Compounds Bearing or not the Corresponding Chemical Substituent	Normalized Negative Impact of the Structural Descriptor, %	Normalized Positive Impact of the Structural Descriptor, %
α-CH_2_	**2 (1)**	−2.1	-	43
α-CH_2_-CH_3_	**3 (1)**	−1.9	-	39
α-CH_2_-CH_2_-CH_3_	**4 (1)**	−2.0	-	41
α-C=C	**5(4), 13(14)**	−2.3	-	47
β-C=C	**6(4)**	+0.6	12	-
α-C=O	**7(2), 8 (3), 9(4), 12(10)**	+4.8	98	-
β-COOH	**10 (3), 12 (8)**	+1.6	33	-
γ-COOH	**14 (4)**	+0.8	16	-
α-OH	**11 (10)**	+0.5	10	-
α-*O*-4/β-5	**15 (4)**	+1.5	31	-
β-*O*-4 ether linked	**18 (9), 19 (8)**	+1.4	29	-
biphenyl (5-5)	**16 (6), 17 (7)**	−0.7	-	14

* model structures are depicted in Figure 1. Parent basic lignin structures are presented in parentheses.

**Table 3:** Characterization of the lignin samples in terms of their composition, molecular weight distribution, functionality, and antioxidant activity.

Sample	Lignin Content, % *	OCH_3_, %	M_w_, Da	Phenolic OH/100 Phenyl Propane Units (PPU)	Relative Content of α-Carbonyl group in the Phenyl-Propane Units, % **	Number of Scavenged DPPH^•^ Radicals Per OH_phen_ (RDI)
Flax soda lignin	94.5 ± 0.5	8.1 ± 0.1	8358	26	9.1 ± 0.1	1.33 ± 0.07
Flax soda lignin CH_2_Cl_2_ fraction	95.4 ± 0.4	6.5 ± 0.1	847	33	22.3 ± 0.1	0.71 ± 0.04
Black alder soda lignin	74.0 ± 0.7	11.2 ± 0.1	7617	48	32.5 ± 0.1	0.77 ± 0.03
Black alder soda lignin, CH_2_Cl_2_ fraction	99.2 ± 0.7	24.0 ± 0.2	638	88	49.5 ± 0.1	0.69 ± 0.03
Ash-tree soda lignin	92.0 ± 0.9	16.3 ± 0.1	4505	47	21.7 ± 0.1	1.01 ± 0.05
Ash-tree soda lignin CH_2_Cl_2_ fraction	98.2 ± 0.5	13.3 ± 0.1	818	85	39.9 ± 0.1	0.72 ± 0.04
TBHQ	-	-	-	200	-	1.31 ± 0.05
Irganox	-	-	-	100	-	1.31 ± 0.07

* based on the results of Py-GC/MS analysis of lignin-derived and carbohydrate-derived products. ** Relative content (%) of structures containing α-carbonyl groups in all lignin-derived products, according to results of the Py-GC/MS analysis.

**Table 4:** Relative content of the phenolic units conjugated with α-carbonyl group given with respect to the total OH phenolic and antioxidant activity of lignins.

Sample	Relative Content of the Phenolic Units Conjugated with α-Carbonyl Group, vs. Total Phenolic Groups, %	Number of Scavenged DPPH^•^ Radicals Per One Phenolic OH Group
Flax soda lignin CH_2_Cl_2_ fraction	14.0	0.71 ± 0.04
Flax soda lignin CH_2_Cl_2_ fraction modified	6.0	2.05 ± 0.8
Black alder soda lignin CH_2_Cl_2_fraction	36.5	0.69 ± 0.03
Black alder soda lignin CH_2_Cl_2_fraction, modified	13.9	1.80 ± 0.02
Ash-tree soda lignin CH_2_Cl_2_ fraction	25.6	0.72 ± 0.04
Ash-tree soda lignin CH_2_Cl_2_fraction, modified	11.3	1.26 ± 0.07
TBHQ	-	1.31 ± 0.05
Irganox 1010	-	1.31 ± 0.07
